# HOW DO EDUCATION AND SOCIAL MEDIA USE MATTER FOR ADVANCE CARE PLANNING AMONG CHINESE OLDER ADULTS?

**DOI:** 10.1093/geroni/igad104.3396

**Published:** 2023-12-21

**Authors:** Yaolin Pei, Xiang Qi, Zheng Zhu, Wei Zhang, Ruey-Ming Tsay, Bei Wu

**Affiliations:** New York University, New York City, New York, United States; New York University, New York City, New York, United States; New York University, New York City, New York, United States; University of Hawaiʻi at Mānoa, Honolulu, Hawaii, United States; Tunghai University, Taichung, Taichung, Taiwan (Republic of China); New York University, New York City, New York, United States

## Abstract

In general, the rate of advance care planning (ACP) engagement is low in Chinese societies globally. Few studies have compared the ACP engagement among Chinese in different regions. This study aims to examine and compare the associations between education, social media use, and advance care planning (ACP) discussion among Chinese older adults in mainland China (Wuhan), Taiwan (Taichung), and United States (Honolulu). Community-dwelling older adults ≥ 55 were recruited from 2017 to 2018. The ACP discussion rate in Wuhan, Taichung, and Honolulu were 15.2%, 19.2%, and 31.3%, respectively. Logistic regression models reveal that education was positively associated with ACP discussion in Taichung and Honolulu. Social media use was positively associated with ACP discussions in Wuhan and Honolulu, and it attenuated the association between education and ACP discussion in Honolulu. The present study contributes to previous research by comparing the association between education, social media use, and ACP discussion in different settings within the same ethnicity. Practice interventions may need use a social media as a tool to promote advance care planning among Chinese older adults where the palliative care is in the initial stage. Form a policy standpoint, it is important to develop palliative care while promoting advance care planning.

